# The hirudin-like factors HLF3 and HLF4—hidden hirudins of European medicinal leeches

**DOI:** 10.1007/s00436-020-06697-1

**Published:** 2020-05-04

**Authors:** Christian Müller, Phil Lukas, Dana Sponholz, Jan-Peter Hildebrandt

**Affiliations:** Animal Physiology and Biochemistry, Zoological Institute and Museum, University of Greifswald, Felix-Hausdorff-Str. 1, 17489 Greifswald, Germany

**Keywords:** Hirudin, Hirudin-like factors, Blood coagulation, Medicinal leeches

## Abstract

The hirudin-like factors 3 (HLF3) and 4 (HLF4) belong to a new class of leech-derived factors and are present in specimens of the three European medicinal leeches, *Hirudo medicinalis*, *Hirudo verbana*, and *Hirudo orientalis*, respectively. Here we describe the functional analysis of natural and synthetic variants of HLF3 and HLF4. Whereas the natural variants display only very low or no detectable anti-coagulatory activities, modifications within the N-termini in combination with an exchange of the central globular domain have the potency to greatly enhance the inhibitory effects of respective HLF3 and HLF4 variants on blood coagulation. Our results support previous observations on the crucial importance of all parts (both the N- and C-termini as well as the central globular domains) of hirudin and HLF molecules for thrombin inhibition.

## Introduction

The saliva of hematophagous leeches comprises a complex mixture of bioactive molecules (Ascenzi et al. 1995; Baskova and Zavalova 2001; Baskova et al. 2008; Hildebrandt and Lemke 2011). To date, merely a handful of these factors are functionally characterized and only the thrombin-inhibitor hirudin found its way from bench to bedside (Becker and Cannon 1994; Greinacher and Warkentin 2008). The hirudin-like factors (HLFs) represent a recently described subclass of compounds derived from the salivary glands of medicinal leeches of the genera *Hirudo* and *Hirudinaria* (Müller et al. 2016; Müller et al. 2017). HLFs comprise structural features that are characteristic for hirudins (e.g., six cysteine residues within a central globular domain and a common gene structure composed of four exons and three introns) but may considerably differ in biochemical properties like molecular weight (MW) and isoelectric point (pI value). In previous works, we have described the purification and functional characterization of HLF1 (originating from *Hirudo medicinalis*) (Müller et al. 2016) and HLF5, 6, and 8 (originating from *Hirudinaria manillensis*) (Lukas et al. 2019), respectively. While HLF1 and HLF6 did not exhibit any measurable anti-coagulatory activities, both HLF5 and HLF8 did.

Natural variants of HLF1 could be identified in specimens of *Hirudo medicinalis*, *Hirudo verbana*, and *Hirudo orientalis*, respectively (Müller et al. 2017). The several variants of HLF1 differ in the length of the C-terminal tail and in the actual number of acidic amino acid residues (ranging from 13 to 16). In addition, they differ in the number (4 or 5) and the composition of the N-terminal amino acid residues (Müller et al. 2017). Strikingly, HLF1 variants with the N-terminal five amino acid residues IVYGP (HLF1V and HLF1long) exhibited very high anti-coagulatory activities comparable to hirudins. In contrast, variants with either four (IYGP, HLF1) or different five N-terminal amino acid residues, specifically IDYEP (HLF1D), had only very low or no detectable activities (Müller et al. 2020). These results were in very good accordance with examinations of the N-termini of hirudins like HV1 of *Hirudo medicinalis* (Betz et al. 1992; Wallace et al. 1989; Lazar et al. 1991) or in HM1 of *Hirudinaria manillensis* (De Filippis et al. 1995, 1998). HLF2 could not yet be successfully purified but almost completely precipitated during dialysis. However, the construction and functional characterization of hybrid variants of HLF1 and HLF2 bypassed this limitation and subsequently revealed strong evidence for the crucial importance of the central globular domain of HLFs on the anti-thrombin activity of the whole molecule as well. The central globular domain of HLF1 enabled thrombin-inhibitory potency, whereas the central globular domain of HLF2 did not (Müller et al. 2020). In the current work, we wanted to apply the same strategy and technical approach to analyze and functionally characterize the hirudin-like factors HLF3 and HLF4.

HLF3 and HLF4 were identified in specimens of European medicinal leeches. HLF3 occurs in two different splice variants (short form: HLF3s or long form: HLF3l), whereas for HLF4, two different splice variants (HLF4a and HLF4b) could be predicted but only mRNA for HLF4a has been found yet (Müller et al. 2017). In both cases, the different splice events occur at the junction between the third exon and the fourth exon and hence do not affect the N-termini or the central globular domains but change the amino acid composition and the lengths of the C-terminal tails. HLF3s and HLF3l predominantly differ in the length of the C-terminal tail and hence the molecular masses (4.17 kDa vs. 6.06 kDa), but the isoelectric points (pI values) are almost identical (9.38 vs. 9.27). The N-terminal five amino acid residues (IVFKP) and the central globular domain of HLF3 are almost identical to the one of HLF2. In contrast, HLF4a and HLF4b differ both in length and composition of the C-terminal tail and hence the pI values (8.30 vs. 4.46), but only slightly in molecular masses (5.30 kDa vs. 5.80 kDa). Interestingly, HLF3l and HLF4b comprise acidic C-terminal tails, a characteristic structural feature of hirudins (Dodt et al. 1984; Scacheri et al. 1993). The N-terminal five amino acid residues of HLF4 (IDYEP) are identical to HLF1D, a natural HLF1 variant without thrombin-inhibitory activity. The central globular domain of HLF4 is similar in size to HLF2 and HLF3 (length of 30 amino acid residues) but is slightly more acidic (pI value of 6.10).

Neither HLF3 nor HLF4 has been purified and functionally tested so far. Thus, the first aim of the present study was closing this gap. Furthermore, we wanted to elucidate the effects of alterations within the N-termini of HLF3 and HLF4 and finally investigate the effects of exchanges of the central globular domains on potential anti-coagulatory and thrombin-inhibitory activities of the respective synthetic HLF3 and HLF4 variants.

## Materials and methods

### Genotyping of animals and tissue preparation

The biological material used in this study (specimen of *Hirudo medicinalis*, *Hirudo verbana*, and *Hirudo orientalis* and salivary gland preparations) was already described by Müller et al. (2016 and 2017). Species identity was confirmed by visual inspection (body coloration pattern) and molecular genotyping. In detail, partial sequences of the internal transcribed spacer 2 (ITS2) as chromosomal marker and the cytochrome c oxidase subunit I gene (*coiI*) as mitochondrial marker, respectively, were determined and compared with database entries. All sequences were deposited in GenBank and obtained the accession numbers KR066919-KR066923 and KR066924-KR066928 (*Hirudo medicinalis*), KX215696-KX215698 and KX215704-KX215706 (*Hirudo verbana*), and KX215699-KX215701 and KX215707-KX215709 (*Hirudo orientalis*), respectively.

### Expression and purification of His-tagged hirudins and HLFs

The procedure to clone cDNAs encoding HLFs and hirudins, to express and purify the respective proteins, was previously described in detail (Müller et al. 2016). Briefly, we applied a system developed by Qiagen (Hilden, Germany). The pQE30Xa vector encodes a factor Xa protease recognition site between the His-tag coding region on the 5′ side and the multiple cloning site on the 3′ side. Factor Xa protease treatment cleaves off the His-tag and results in a recombinant protein that is free of any vector-derived amino acids at the N-terminus.

Partial cDNAs of HLFs were cloned into pQE30Xa in a way that the first amino acid of the respective HLF (without the signal sequence) was located directly adjacent to the factor Xa protease cleavage site. The cDNA sequences of interest were amplified using appropriate primer pairs and Q5® High-Fidelity DNA Polymerase (New England Biolabs, Frankfurt a. M., Germany). For the expression pQE30Xa, clones containing inserts encoding the HLF variants of interest were transformed into appropriate *Escherichia coli* strains. Two flasks, each containing 500 ml of lysogeny broth (LB) medium with ampicillin, were inoculated with 10 ml of a preculture. From the start of inoculation, optical densities were determined in a regular frequency. At an OD_600_ = 0.5, the expression of hirudin and HLF variants was induced by adding IPTG to a final concentration of 1 mmol/l. After 4 h of expression, cells were harvested, the pellet was carefully resuspended in binding buffer (20 mmol/l Tris/HCl, 500 mmol/l NaCl, 5 mmol/l imidazole, pH 7.9), and the cells were sonicated using a Sonopuls homogenizer (Bandelin, Berlin, Germany). After centrifugation for 1 h at 4 °C and 4500 rpm (appr. 3900×*g*) in a Labofuge 400R (Thermo Scientific, Schwerte, Germany), the supernatant was loaded on a self-packed column containing Ni-iminodiacetic acid (IDA) His-Bind® resin (Merck, Darmstadt, Germany). Washing and elution steps were performed as recommended by the manufacturer of the resin. Equal volumes of every fraction were analyzed by sodium dodecyl sulfate polyacrylamide gel electrophoresis (SDS-PAGE) on 20% gels. Prior to the treatment with factor Xa protease, fractions of interest were dialyzed twice for 24 h at 4 °C against a 100-fold excess of reaction buffer (20 mmol/l Tris/HCl, 100 mmol/l NaCl, 2 mmol/l CaCl_2_, pH 8.0) in a dialysis membrane with a molecular weight cutoff (MWCO) of 5000 (Roth, Karlsruhe, Germany). The final volume was approximately 10 ml.

### Factor Xa protease treatment and purification

The treatment of fusion proteins containing the factor Xa protease recognition sequence consisted of three steps: (1) factor Xa protease cleavage, (2) removal of factor Xa protease, and (3) clean up of the digested protein. All steps were performed as recommended by the manufacturer (Qiagen, Hilden, Germany). Purity of recombinant hirudins and HLFs was confirmed by SDS-PAGE on 20% gels. Molar concentrations of protein solutions were calculated by dividing the absorbance at 280 nm by the molar absorption coefficient according to the equation *ε* = (nW × 5500) + (nY × 1490) + (nC × 125) (Gill and von Hippel 1989; Pace et al. 1995).

### Blood coagulation assays

To verify the biological activity of purified HLFs, we performed the thrombin time test (TT; reference range 16.8–21.4 s) using a BFT II analyzer (Siemens Healthcare, Erlangen, Germany). All steps followed the instructions outlined by the manufacturer. For the coagulation tests, all protein samples were diluted with dialysis buffer to reach final concentrations in the reaction assays of 3.2 μmol/l or 0.32 μmol/l, respectively. The desired amount of substrate was directly transferred into the cuvette immediately before the plasma was added. Dade® Ci-Trol® 1 (Siemens Healthcare, Erlangen, Germany) was used as standardized human plasma. The incubation of reaction mixtures was carried out at 37.4 °C. Measurements that lasted up to 300 s were stopped and declared as a complete inhibition of clot formation.

### Generation of hybrid and synthetic HLF variants

The hybrid variants of HLF3 and HLF4 were generated using the gene synthesis service of Synbio Technologies (Monmouth Junction, NJ, USA). All other HLF variants were generated by PCR using appropriate primers to incorporate alterations in the nucleotide sequences that lead to the desired alterations in the amino acid sequences. Table 1 summarizes the origin (natural or synthetic) of all factors that are described and analyzed in the study.

**Table 1:**
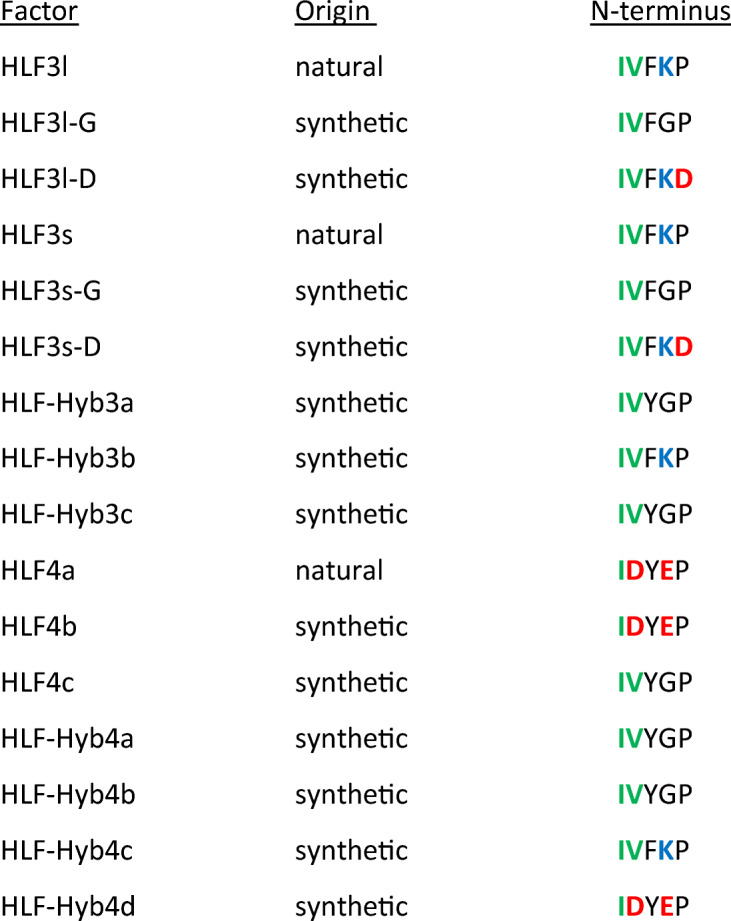
Origin (natural or synthetic) and N-terminal amino acid sequences of all factors described and analyzed in the study

Acidic residues are marked in red, basic residues are marked in blue, and neutral aliphatic residues are marked in green

## Results

The main aim of the present study was to characterize the hirudin-like factors HLF3 and HLF4 in their ability to negatively influence thrombin activity in human plasma and hence the blood coagulation cascade. Both naturally occurring and genetically modified variants of the respective hirudin-like factors were tested in thrombin time coagulation assays, especially synthetic hybrid molecules containing combinations of domains of HLFV1, HLF3, or HLF4.

### Functional analysis of HLF3 variants

**Fig. 1:**
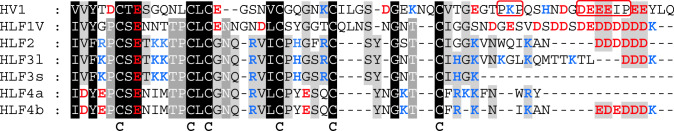
Multiple sequence alignment of hirudin variant HV1 of *H. medicinalis* and HLF variants HLF1V, HLF2, HLF3l, HLF3s, HLF4a, and HLF4b. The alignments were generated using the CLS Sequence Viewer software package v8.0 (CLC bio, Aarhus, Denmark). Black background indicates conserved residues; gray background indicates similar residues. The six conserved cysteine residues giving rise to the three-dimensional structure are marked in bold, acidic amino acid residues are marked in red, and basic amino acid residues are marked in blue. The PKP and DFxxIP motifs are boxed. Abbreviations are used according to the IUPAC code

**Fig. 2:**
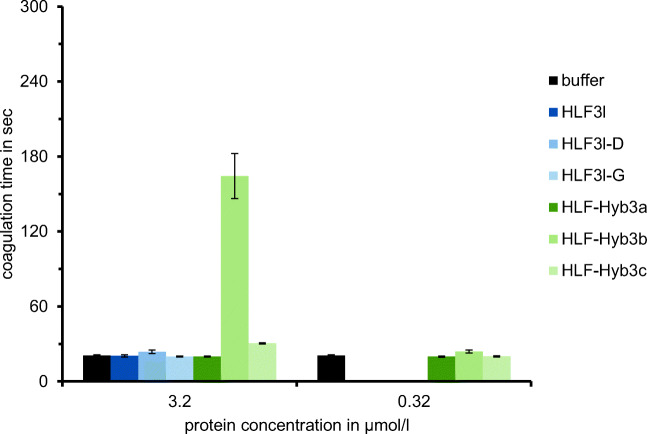
Standard blood coagulation assays using the thrombin time test (TT) of HLF variants HLF3l, HLF3l-G, and HLF3l-D as well as the hybrids HLF-Hyb3a, HLF-Hyb3b, and HLF-Hyb3c. *n* = 3; error bars indicate s.d

The cDNAs of HLF3l and HLF3s are splice variants of the same gene and encode putative proteins with identical N-termini and central globular domains, but different C-terminal tails. Interestingly, the N-termini (IVFKP) are different from both the hirudin variant HV1 (VVYTD) and the hirudin-like factor HLF1V (IVYGP) (see Fig. 1). We constructed variants of both HLF3l and HLF3s and replaced the basic amino acid residue lysine at position 4 with a glycine residue as in HLF1V (HLF3l-G and HLF3s-G) or the proline residue at position 5 with an aspartic acid residue as in HV1 (HLF3l-D and HLF3s-D) (see Table 1). All six variants of HLF3 were expressed, purified, and functionally tested as described in “Materials and methods.” However, none of the variants exhibited anti-thrombin activity at final concentrations of 3.2 μmol/l (or 1.9 μmol/l for HLF3s) (Fig. 2; data not shown for the HLF3s-variants).

As already outlined above, the N-termini and central globular domains of HLF2 and HLF3 are almost identical (Fig. 1; Table 2). According to previous investigations, the presence of the central globular domain of HLF2 renders HLF hybrids unable to inhibit thrombin (Müller et al. 2020). We hence constructed three different hybrids of the highly active thrombin inhibitor HLF1V and HLF3l: HLF-Hyb3a (N- and C-termini of HLF1V fused to the central globular domain of HLF3), HLF-Hyb3b (N- and C-termini of HLF3l fused to the central globular domain of HLF1V), and HLF-Hyb3c (N-terminus and central globular domain of HLF1V fused to the C-terminal tail of HLF3l) (Fig. 4a; Table 1 and Table 3). Again, all three hybrids were expressed, purified, and functionally tested. Whereas HLF-Hyb3a displayed no anti-thrombin activity, HLF-Hyb3b and HLF-Hyb3c clearly did, at least when tested at the high concentration of 3.2 μmol/l (Fig. 2). Hence, integration of the central globular domain of HLF1V converted the previously inactive HLF3l to a thrombin inhibitor. Interestingly, the inhibitory potency of HLF-Hyb3b (containing the N-terminus of HLF3) was much higher compared to HLF-Hyb3c (containing the N-terminus of HLF1V).

**Table 2:** Molecular properties of hirudin variant HV1 and HLF variants HLF1, HLF2, HLF3, and HLF4

**Factor**	**Length C1-C6**	**Acidic/basic**	**pI value**
HV1	34	4/2	4.41
HLF1	34	3/0	3.57
HLF2	30	1/5	8.68
HLF3	30	1/5	8.68
HLF4	30	2/2	6.10
**Factor**	**C-terminal tail**	**Acidic/basic**	**pI value**
HV1	26	8/2	3.94
HLF1	24	13/1	3.26
HLF2	18	6/2	3.85
HLF3l	21	3/6	9.40
HLF3s	4	0/2	8.76
HLF4a	9	0/4	11.10
HLF4b	15	6/4	4.53

C1-C6 indicates the number of amino acid residues between the cysteine residues 1 and 6 (including C1 and C6). C-terminal tail indicates the number of amino acid residues starting immediately after C6. The ratio acidic/basic indicates the number of acidic and basic amino acid residues within the respective region together with the deduced pI value

**Table 3:** Composition and activity of HLF3s/l, HLF4a/b, and HLF3 and HLF4 hybrids

Variant	N-terminus	Central domain	C-terminus	Inhibitory activity
HLF3l	HLF3	HLF3	HLF3l	No
HLF3s	HLF3	HLF3	HLF3s	No
HLF-Hyb3a	HLF1V	HLF3	HLF1	No
HLF-Hyb3b	HLF3	HLF1	HLF3l	Medium
HLF-Hyb3c	HLF1V	HLF1	HLF3l	Low
HLF4a	HLF4	HLF4	HLF4a	Very low
HLF4b	HLF4	HLF4	HLF4b	No
HLF4c	HLF1V	HLF4	HLF4b	No
HLF-Hyb4a	HLF1V	HLF4	HLF1	No
HLF-Hyb4b	HLF1V	HLF1	HLF4b	Low-medium
HLF-Hyb4c	HLF3	HLF1	HLF4b	High
HLF-Hyb4d	HLF4	HLF1	HLF4b	No

### Functional analysis of HLF4 variants

**Fig. 3:**
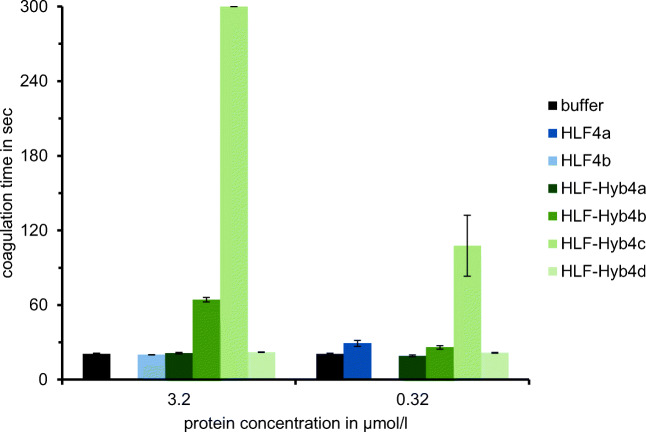
Standard blood coagulation assays using the thrombin time test (TT) of HLF variants HLF4a, HLF4b, and HLF4c as well as the hybrids HLF-Hyb4a, HLF-Hyb4b, HLF-Hyb4c, and HLF-Hyb4d. *n* = 3; error bars indicate s.d

Comparably to the HLF3 gene, the HLF4 gene may encode two different mRNAs/cDNAs as well. Whereas expression of the HLF4a mRNA could already be verified (Müller et al. 2017), attempts to detect the expression of a HLF4b mRNA failed so far, at least in leech salivary gland cells. A putative HLF4b protein comprises structural and biochemical features that resemble hirudins: a pI value of 4.46 and an acidic C-terminal tail. The N-terminus and the central globular domain, however, are distinct from those of HV1 and HLF1V (Fig. 1; Table 2). In addition to HLF4b, we constructed a variant (HLF4c) that comprises the N-terminus of HLF1V. Subsequently, we tried to express, purify, and functionally test HLF4a, HLF4b, as well as HLF4c. For both HLF4a and HLF4c, the procedure turned out to be problematic due to the formation of inclusion bodies (HLF4a) or precipitation of the protein during dialysis (HLF4c). All factors were functionally tested at the highest possible concentrations of 3.2 μmol/l (HLF4b), 0.74 μmol/l (HLF4c), or 0.32 μmol/l (HLF4a). HLF4b and HLF4c (data not shown) did not exhibited anti-thrombin activity, whereas HLF4a had a weak thrombin-inhibitory potency (Fig. 3).

**Fig. 4:**
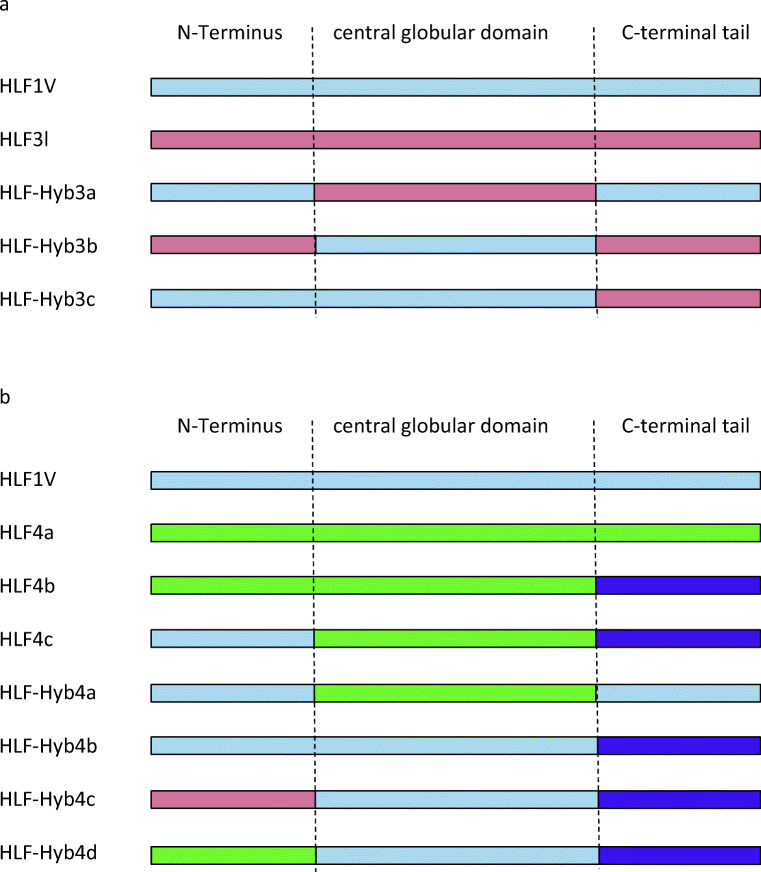
**a** Schematic representation of the N-termini, the central globular domains, and the C-terminal tails of HLF1V (light blue) and HLF3l (light red) and the hybrid variants HLF-Hyb3a, HLF-Hyb3b, and HLF-Hyb3c. **b** Schematic representation of the N-termini, the central globular domains, and the C-terminal tails of HLF1V (light blue), HLF3 (light red), HLF4a (light green), and HLF4b (purple) and the hybrid variants HLF-Hyb4a, HLF-Hyb4b, HLF-Hyb4c, and HLF-Hyb4d

To verify the influence of the central globular domain of HLF4, we constructed the following hybrid variants of HLF4, HLF3, and HLF1V: HLF-Hyb4a (N- and C-termini of HLF1V fused to the central globular domain of HLF4), HLF-Hyb4b (N-terminus and central globular domain of HLF1V fused to the C-terminal tail of HLF4b), HLF-Hyb4c (N-terminus of HLF3 fused to the central globular domain of HLF1V and the C-terminal tail of HLF4b), and finally HLF-Hyb4d (N-terminus of HLF4 fused to the central globular domain of HLF1V and the C-terminal tail of HLF4b) (Fig. 4b; Table 1 and Table 3). Again, all hybrid factors were expressed, purified, and functionally tested. Neither HLF-Hyb4a nor HLF-Hyb4d displayed any anti-thrombin activities. In contrast, both HLF-Hyb4b and HLF-Hyb4c did (Fig. 3). Strikingly, the thrombin-inhibitory potency of HLF-Hyb4c (containing the N-terminus of HLF3) was much higher compared to that of HLF-Hyb4b (containing the N-terminus of HLF1V), an observation that is in line with the data obtained for HLF-Hyb3b and HLF-Hyb3c (see above). Like for HLF3 (see above), the central globular domain of HLF4 prevents any anti-thrombin activity of respective factors.

## Discussion

In previous investigations, we have analyzed and functionally characterized the hirudin-like factors HLF1 and HLF2 as well as natural and synthetic hybrid variants thereof (Müller et al. 2016, 2020). A similar approach was applied to analyze and functionally characterize the hirudin-like factors HLF3 and HLF4 too. As a result, we expected a more detailed impression on the importance of the three distinct functional units of all hirudin and HLF molecules (namely the N-terminus, the central globular domain, and the C-terminal tail) on their respective thrombin-inhibitory and hence anti-coagulatory capacities.

Both HLF3 and HLF4 may occur in two different splice variants each, namely HLF3l/HLF3s and HLF4a/HLF4b. None of these factors displayed a significant thrombin-inhibitory potency when tested in the thrombin-time assay. The low, but detectable thrombin inhibition by HLF4a cannot be satisfactorily explained at the moment and might be an artifact due to the fact that it was impossible to purify the factor by the standard procedure, but only following a different protocol (e.g., including resolubilization of inclusion bodies under denaturating conditions followed by a refolding step). The hirudin-like factors HLF1D (Müller et al. 2020) and HLF-Hyb4d (Fig. 3) displayed no inhibitory activity on thrombin despite “suitable” central globular domains and C-terminal tails. The lack of inhibitory activities in both cases is hence most likely caused by the N-terminal five amino acid residues IDYEP. The same residues are present in HLF4a as well and should therefore consequently prevent its anti-thrombin activity.

But even with the “more suitable” N-terminus of HLF1V (IVYGP), neither HLF3l-G nor HLF4c displayed any anti-thrombin activity (Figs. 2 and 3). Thrombin inhibition was only achieved with constructs containing the central globular domain of HLF1 instead of those of HLF3 and HLF4, giving rise to the hybrid factors HLF-Hyb3b/HLF-Hyb3c and HLF-Hyb4b/HLF-Hyb4c, respectively (Figs. 2 and 3). These observations strongly confirm the hypothesis (formulated based on the analysis of HLF1/HLF2-hybrids; Müller et al. 2020) that not only the N- and C-termini but also the central globular domains of hirudins and hirudin-like factors are of crucial importance for the ability of the respective molecules to inhibit thrombin.

However, the exact composition of the N- and C-termini matters as well. The N-terminus of HLF1V (IVYGP) works well (HLF1V has thrombin-inhibitory potency which is almost as high as those of the hirudin variants HV1 and HV2; Müller et al. 2020), but the N-termini of HLF2 (IVFRP) or HLF3 (IVFKP) seem to work even better: the hybrid variants comprising the N-terminus of HLF1V (HLF-Hyb2a, HLF-Hyb3c and HLF-Hyb4b) consistently have lower inhibitory potencies compared to the otherwise identical hybrid variants comprising the N-termini of HLF2 or HLF3 (HLF-Hyb2b, HLF-Hyb3b, and HLF-Hyb4c). One might speculate that a variant of HLF1 comprising the N-terminus of HLF2 or HLF3 would be kind of a “super inhibitor” for thrombin.

The C-terminal tail of HLF1, on the other hand, is very likely much more effective compared to the C-termini of both HLF3l and HLF4b. Even at the low concentration of 0.32 μmol/l in the thrombin time assay HLF1V completely blocked the activity of hirudin (Müller et al. 2020). The inhibitory potencies of both HLF-Hyb3c and HLF-Hyb4b were much lower (Figs. 2 and 3), and this might very well be explained by a combination of length and charge effects of their C-terminal tails. The C-terminal tails of hirudins block the exosite 1, the fibrinogen binding site, of thrombin (Rydel et al. 1991; Maraganore et al. 1989; Fenton 2nd et al. 1991). This effect is mainly due to several ionic and non-ionic interactions between particular amino acid residues in both hirudin and thrombin (Betz et al. 1991b; Huang et al. 2014; Braun et al. 1988). Of particular importance within this context are the numerous acidic amino acid residues (7 out of 29 residues in HV1, 9 out of 27 residues in HM1) (Betz et al. 1991a). HLF1V (13 out of 24) contains even more acidic amino acid residues in its tail. However, as already pointed out, an elongated acidic tail per se does not guarantee a significant inhibitory potency of a hirudin or a hirudin-like factor but needs a functional N-terminus and an appropriate central globular domain as well. We therefore concluded that the C-terminal tails are much less constrained compared to the N-termini and the central globular domains with respect to determining thrombin-inhibitory potency of the resulting molecules. However, an overall acidic character of the C-terminus seemed to be essential for thrombin inhibiting potency of the final construct (Müller et al. 2020). The C-terminal tails of HLF3l and HLF4b almost perfectly fit into this scheme. Both are shorter compared to HV1 and HLF1 but contain several charged amino acid residues (Table 2). However, whereas the tail of HLF4b is shorter, but indeed acidic and negatively charged (pI value of 4.53), the tail of HLF3l is longer, but basic and positively charged (pI value of 9.40). The shorter and acidic tail of HLF4b resulted in a somewhat higher thrombin-inhibitory potency compared with HLF3l which has the longer, but positively charged tail (compare the activities of HLF-Hyb3b and HLF-Hyb4c; Figs. 2 and 3). We concluded that an overall acidic character of the C-terminal tail of hirudins/hirudin-like factors is advantageous, but not absolutely mandatory for anti-thrombin activity, as long as several charged including some acidic amino acid residues are present.

The biological functions of the naturally occurring HLF3s/HLF3l and HLF4a/HLF4b remain obscure. Whatever their real targets may be (if there are any at all), both genes have very likely evolved from a common ancestor that encoded a hirudin-like thrombin inhibitor. The general potency of hirudin-like factors to act as thrombin inhibitors like hirudin is obvious: HLF1, HLF2, HLF3, and HLF4 can quite easily be transformed from non-inhibitors into inhibitors by exchanging certain domains. The molecular techniques that we have applied to construct hybrids (e.g., gene synthesis or introduction of point mutations) may easily be mirrored by natural processes. Gene re-arrangement by recombination (Roberstson 1982) and exon shuffling (Wang et al. 2016) are only two of various potential mechanisms that are present in the genetic toolbox of every organism. The multiplicity of hirudins and hirudin-like factors in both European and Asian medicinal leeches may hence serve as excellent examples to illustrate and evaluate the mechanisms and outcomes of gene and genome evolution processes in general.
